# A painful twist: Wandering spleen with torsion and infarction: A case report

**DOI:** 10.1016/j.ijscr.2025.111391

**Published:** 2025-04-28

**Authors:** Abdelrahman S. Elnour, Adam Yagoub, Ahmed Saeed, Faisal Nugud, Ahmed A. Alshaikh

**Affiliations:** a National Centre for Pediatric Surgery, Sudan; b Federal Ministry of Health, Sudan; c Department of Surgery, Faculty of Medicine, University of Gezira, Wad Madani, Sudan

**Keywords:** Wandering spleen, Splenic torsion, Splenic infarction, Splenectomy, Case report

## Abstract

**Introduction:**

Wandering spleen is a rare condition characterized by abnormal spleen mobility due to defects in its supporting ligaments. Delayed management can lead to severe complications such as torsion and infarction, making early diagnosis and surgical intervention crucial for preventing adverse outcomes.

**Case presentation:**

A 16-year-old girl presented with recurrent episodes of severe left hypochondrial pain, which worsened over six months, accompanied by occasional vomiting. Physical examination revealed a tender, palpable spleen extending from the left hypochondrium to the left iliac fossa. Imaging studies, including abdominal ultrasound and contrast-enhanced CT scan, revealed an enlarged spleen with signs of pedicle torsion and infarction, confirming a diagnosis of wandering spleen with vascular compromise. An emergency splenectomy was performed, revealing significant splenic enlargement with torsion and infarction, with no ligamentous attachments. The patient recovered uneventfully and received a pneumococcal vaccine along with long-term antibiotic prophylaxis prior to discharge.

**Discussion:**

Wandering spleen often presents with nonspecific symptoms, leading to misdiagnosis or delayed treatment. Diagnostic imaging is essential for accurate identification. Management typically involves surgery, with splenopexy preferred when feasible and splenectomy reserved for cases with complications.

**Conclusion:**

Wandering spleen, though rare, requires prompt recognition and management to prevent complications like torsion and infarction.

## Highlights

• Wandering spleen is a rare but serious condition, posing risks of torsion and infarction.
• Progressive abdominal pain with a mobile spleen warrants urgent evaluation.
• Ultrasound and CT imaging are critical for timely diagnosis.
• Early surgical intervention is key, splenopexy preserves the spleen when feasible, while splenectomy is required for complications.

## 1 Introduction

The spleen is normally located in the left hypochondrium and is held in position by several suspensory ligaments [1,2]. Wandering spleen is a rare condition characterized by excessive splenic mobility due to congenital maldevelopment or elongation of its supporting ligaments. It typically presents with abdominal pain, which may be acute or intermittently chronic due to splenic torsion. In some cases, it manifests as a painless, palpable abdominal mass. This condition is most commonly observed in children and young adults, particularly women of reproductive age, with an incidence of less than 0.2 % [2,3].

Identifying wandering spleen in children can be challenging due to nonspecific symptoms. However, diagnostic tools such as plain X-rays, Doppler ultrasound, computed tomography (CT), magnetic resonance imaging (MRI), scintigraphy, and splenic angiography are useful for confirmation [[4], [5], [6]].

The ideal treatment for wandering spleen involves surgical intervention, with splenopexy being the preferred choice in the absence of infarction, splenomegaly, or hypersplenism. However, splenectomy is necessary if these complications are present [7,8].

Despite its rarity, wandering spleen can lead to life-threatening complications such as splenic torsion and infarction, requiring prompt diagnosis and management. Herein, we report a case of a 16-year-old girl who presented with acute abdominal pain and was ultimately diagnosed with wandering spleen, pedicle torsion, and splenic infarction. This patient is unique due to delayed presentation, which posed additional diagnostic and management challenges. This report aims to highlight the diagnostic challenges of wandering spleen in pediatric patients and underscores the vital role of imaging and timely surgical intervention. This case report is reported in line with SCARE criteria [9].

## 2 Case presentation

A 16-year-old girl presented to our hospital with recurrent episodes of sudden, severe left hypochondrial pain that began six months earlier. The pain was occasionally accompanied by vomiting, was unresponsive to analgesia, and resolved spontaneously. There were no symptoms of intestinal obstruction. Over time, the pain became more frequent and severe, prompting referral for surgical evaluation.

On physical examination, the patient appeared ill, in pain, and tachycardic. There were no signs of respiratory compromise. Abdominal examination revealed a palpable, tender spleen extending from the left hypochondrium to the left iliac fossa. Laboratory tests of full blood count, renal and liver function were within normal limits.

**Fig. 1:**
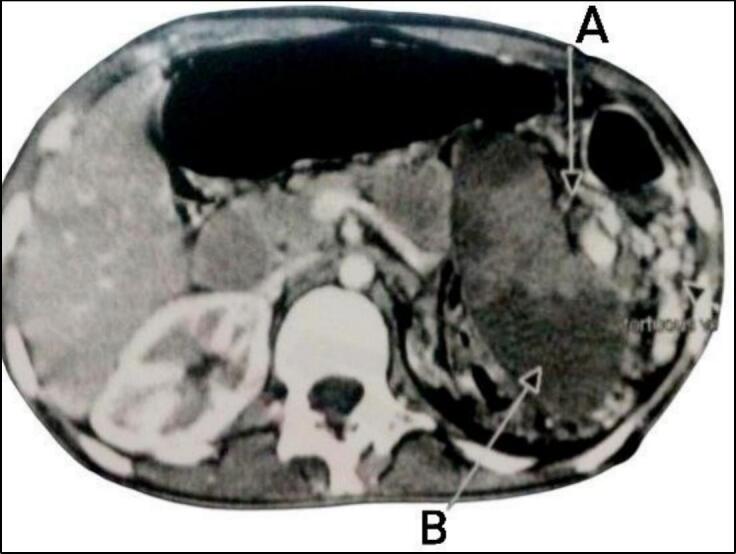
Selected cut of contrast-enhanced CT of the abdomen showing an enlarged spleen with torsion of its pedicle (A) and heterogeneous hypodense areas that consistent with infarction (B).

Abdominal ultrasound showed an enlarged spleen, with reduced perfusion in focal areas, raising suspicion of torsion. A contrast-enhanced CT scan confirmed an enlarged spleen measuring 21 cm, extending into the left iliac fossa, with evidence of pedicle torsion and lateral displacement of the hilum. Heterogeneous hypodensity in the proximal spleen suggested infarction. Additionally, the left kidney appeared atrophic, while the right kidney showed compensatory hypertrophy (Fig. 1).

**Fig. 2:**
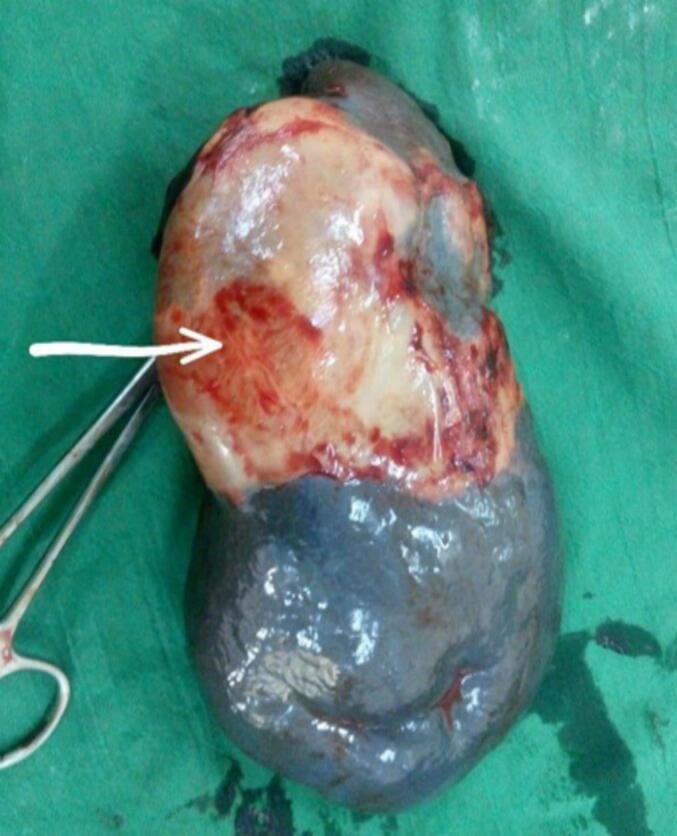
Splenectomy specimen showing a large spleen with a gray area of infarction (arrow).

A diagnosis of wandering spleen with torsion and infarction was made, and the patient was prepared for urgent splenectomy. Intraoperatively, the spleen was significantly enlarged, with torsion, a laterally facing pedicle, and no ligamentous attachments. It was anchored to the lateral abdominal wall by a fibrovascular band at the infarcted area. The splenectomy was successfully performed (Fig. 2). The patient received a pneumococcal vaccine on postoperative day one and was discharged in good condition after five days, with a regimen of long-term antibiotic prophylaxis.

## 3 Discussion

Wandering spleen is a rare but significant condition that can lead to serious complications if not diagnosed and treated promptly. In this case study, we examined a 16-year-old girl who presented with recurrent severe left hypochondrial pain, ultimately diagnosed with wandering spleen complicated by pedicle torsion and splenic infarction. This case highlights several key points regarding the clinical presentation, diagnostic challenges, and management of wandering spleen.

Qian et al. [8] analysed 32 case reports and case series involving 37 wandering spleen cases (25 females and 12 males) aged 12–18 years. Their findings indicated no sex-based differences in prevalence under the age of 10 years, but a higher prevalence among females in the 12–18 age group [8].

The initial symptoms of wandering spleen can be nonspecific, often leading to misdiagnosis or delayed treatment [10]. The patient in this report was referred to our facility six months after experiencing recurrent abdominal pain associated with vomiting, which could easily be attributed to other gastrointestinal issues. This emphasizes the importance of considering wandering spleen in differential diagnoses for young patients presenting with unexplained abdominal pain, particularly when accompanied by a palpable mass. Increasing awareness among clinicians about this rare condition is crucial for early recognition and timely intervention, which can prevent severe complications such as splenic torsion and infarction.

Laboratory investigations in patients with wandering spleen are typically normal and nonspecific. However, some cases may show abnormalities such as leucocytosis, thrombocytopenia, neutrophilia, mild pancytopenia, elevated serum amylase and lipase levels, or increased C-reactive protein levels [9]. In our case, the full blood count, as well as renal and liver function tests, were within normal limits, highlighting the relatively low diagnostic utility of laboratory investigations in this condition.

Diagnostic imaging plays a crucial role in identifying wandering spleen. Imaging methods such as plain X-ray, Doppler ultrasonography (USG), computed tomography (CT), and magnetic resonance imaging (MRI) are typically used to confirm the diagnosis [9,11]. Contrast-enhanced ultrasound (CEUS) has also been explored for diagnosing wandering spleen, with images displaying the absence of the spleen in the left upper quadrant and a soft tissue mass in other abdominal or pelvic areas [12]. Despite the various imaging techniques available, CT remains the preferred method for definitive diagnosis due to its high sensitivity in detecting splenic pedicle torsion [13,14]. In our case, a contrast-enhanced CT scan was instrumental in revealing the enlargement of the spleen and signs of torsion and infarction.

The management of wandering spleen primarily involves surgical intervention. While splenopexy is preferred when feasible, splenectomy may be necessary in the presence of complications such as infarction or torsion, or when splenopexy is not possible [14,15]. In our case, splenectomy was performed due to torsion and infarction. This underscores the importance of early diagnosis and prompt surgical intervention, which are essential for preserving spleen function and improving surgical outcomes. Timely management also helps prevent both short- and long-term complications of splenectomy, including haemorrhage and sepsis.

Overwhelming post-splenectomy infection (OPSI) is the major long-term complication of splenectomy, which can rapidly progress from flu-like symptoms to septic shock. The highest risk occurs within the first 3 years post-splenectomy, although the risk remains elevated throughout life. Children, trauma patients, individuals with haematological malignancies, and immunocompromised patients are at greater risk. Despite adequate treatment, the mortality rate for OPSI remains high, underscoring the need for preventive strategies in managing splenectomised patients [16]. In this case, our patient received a pneumococcal vaccine to reduce the risk of overwhelming infections. However, other vaccines, including Haemophilus influenzae and meningococcal vaccines, which are part of the standard post-splenectomy protocol, were unfortunately unavailable in our setting. This highlights the significant challenges faced by patients in low-income countries and underscores the urgent need for long-term postoperative management strategies that address gaps in vaccine availability and ensure comprehensive follow-up care.

## 4 Conclusion

Wandering spleen, though rare, requires prompt recognition and management to prevent complications like torsion and infarction. This case underscores the diagnostic challenges posed by nonspecific symptoms in pediatric patients and the importance of imaging for accurate diagnosis. Clinicians should maintain a high index of suspicion for wandering spleen in young patients with abdominal pain and palpable masses. Timely surgical intervention is critical for optimal outcomes.

## CRediT authorship contribution statement

Abdelrahman S. Elnour: Designed the study concept, treated the patient, collected data, and wrote the manuscript.
Adam Yagoub and Ahmed Saeed: Provided patient treatment and contributed to manuscript revision.
Faisal Nugud and Ahmed A. Alshaikh: Contributed to manuscript revision.

## Consent

Informed consent was obtained from the patients' parent for publication and any accompanying images. A copy of the written consent is available for review by the Editor-in-Chief of this journal on request.

## Ethical approval

Not applicable.

## Guarantor

Abdelrahman S. Elnour.

## Funding

None.

## Declaration of competing interest

None.
